# Factitious Cheilitis: Report of Two Cases

**DOI:** 10.1002/ccr3.71792

**Published:** 2026-01-04

**Authors:** Daniel Mauricio Cuestas Rodriguez, Tatiana Carolina Reyes Vivas, Luis Daniel Pérez Cáceres, Laura Sofía Martínez Martínez, Nahomy Giraldo Mejía, Valentina Alvarez Mengual

**Affiliations:** ^1^ Universidad el Bosque, Simón Bolívar Hospital Bogotá D.C. Colombia; ^2^ Centros Médicos Colsanitas Bogotá D.C. Colombia; ^3^ Fundación Universitaria Sanitas Bogotá D.C. Colombia; ^4^ Fundación Universitaria de Ciencias de la Salud, Hospital de San José Bogotá D.C. Colombia; ^5^ Universidad Antonio Nariño Bogotá D.C. Colombia; ^6^ Universidad Autónoma de Bucaramanga Bucaramanga Colombia

**Keywords:** differential diagnosis, factitious cheilitis, lip lesions, psychiatric comorbidity, psychodermatology, self‐inflicted dermatoses

## Abstract

Factitious cheilitis (FC) is a rare psychodermatologic condition characterized by self‐inflicted trauma to the lips. It is frequently underdiagnosed and often mistaken for infectious or neoplastic processes. We report two cases of young adult female patients with painful verrucous lip lesions. In both cases, initial clinical suspicion included squamous cell carcinoma and pustular cheilitis. Histopathologic findings were nonspecific. Clinical history and behavior patterns revealed self‐inflicted trauma. Both patients were ultimately diagnosed with FC and managed with topical treatment and referral for psychological support. These cases highlight the diagnostic challenges of FC and the importance of recognizing self‐inflicted dermatoses in dermatologic practice. Failure to do so can lead to unnecessary procedures and treatments. FC should be considered in patients with chronic cheilitis and atypical lip lesions, especially in those with psychiatric comorbidities. A multidisciplinary approach is essential for proper diagnosis and management.

## Key Clinical Message

Factitious cheilitis should be considered as a differential diagnosis or suspected in patients with crusted plaques lesions on the lips who have a history of anxiety disorders or behaviors that cause repeated microtrauma in the areas where the lesions appear.

## Graphical abstract

Differential diagnosis of factitious cheilitis. Conditions affecting the upper lip include perioral dermatitis, herpes simplex, allergic contact dermatitis, and syphilis. Disorders involving the lower lip include actinic cheilitis, atopic cheilitis, irritant contact cheilitis, herpes infection, and candidiasis. Conditions affecting both lips include exfoliative cheilitis, candidiasis, severe xerosis, and Munchausen syndrome.

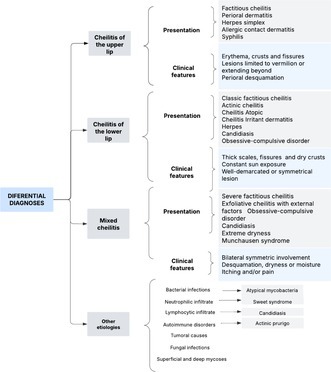

## 1 Introduction

Factitious cheilitis (FC) is an artifact‐related dermatosis characterized by white‐grayish plaques on the vermilion border of the lips, caused by repetitive self‐inflicted trauma. This condition is rare and frequently underdiagnosed, predominantly affecting women and young adults, many of whom have underlying psychiatric histories. Due to its prominent clinical features, diagnosis is often delayed, resulting in repeated biopsies with nonspecific findings and the administration of empirical treatments and unnecessary investigations—leading to significant healthcare costs. We present two cases of young adult women who were admitted to the dermatology department for verrucous lesions involving both lips.

## 2 Case 1

### 2.1 Clinical History/Examination

A 31‐year‐old female patient with a history of extensive longitudinal myelopathy presented with spastic paraparesis and neurological sequelae affecting the gastrointestinal and genitourinary systems.

**FIGURE 1:**
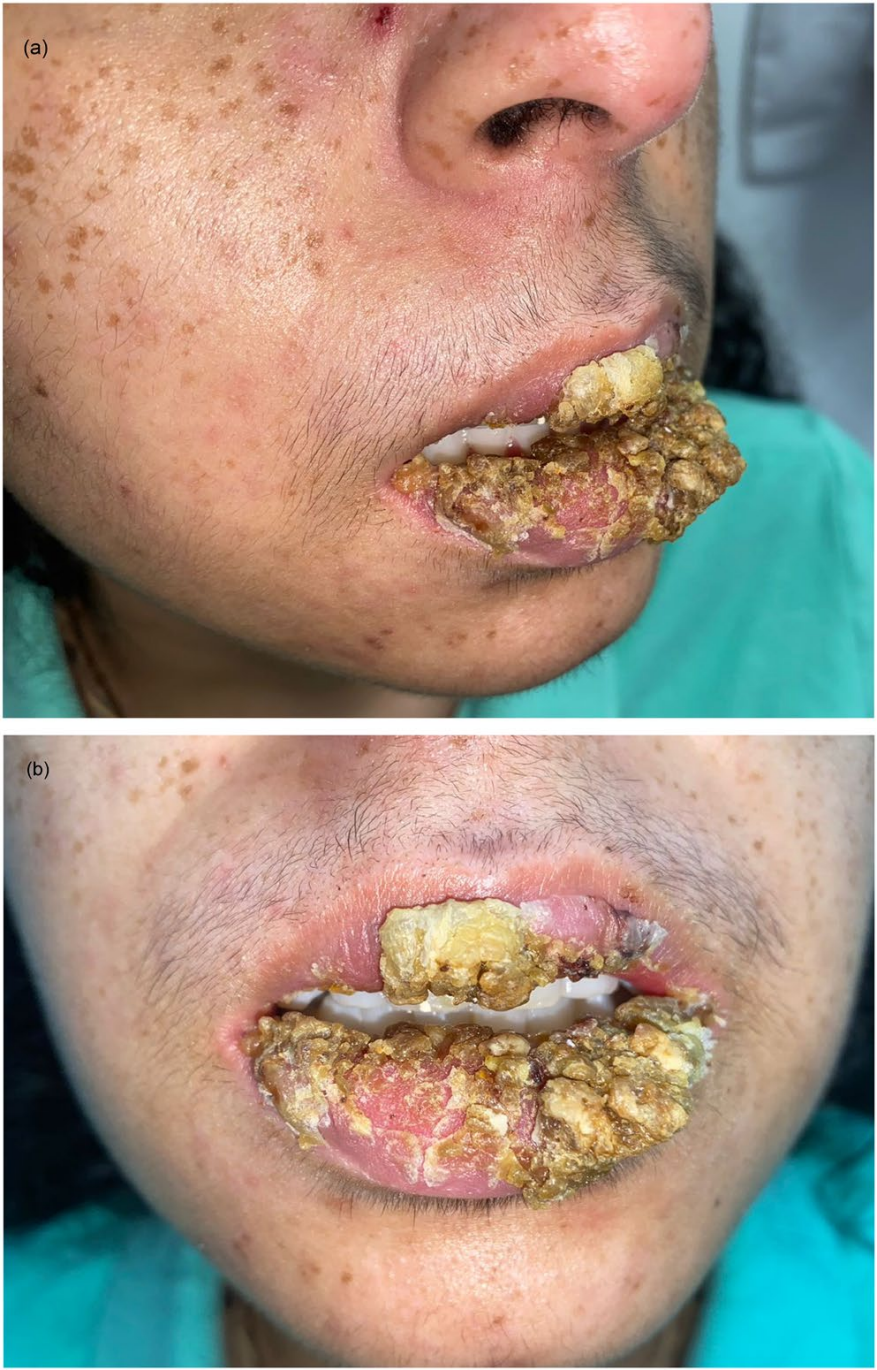
Case 1: (A, B) Multiple hyperkeratotic verrucous plaques on the upper and lower lips.

She was referred to the dermatology department with a 1‐year history of progressively worsening verrucous lesions affecting both the upper and lower lips. The lesions were painful, rapidly growing, and recurrent, with no identifiable trigger despite prior surgical excision. The patient reported significant pain and functional limitation associated with these lesions (Figure 1A,B).

### 2.2 Differential Diagnosis, Investigations, and Treatment

Given the exuberant and keratotic appearance of the lesions, initial clinical considerations included squamous cell carcinoma and epidermodysplasia verruciformis.

A skin biopsy was performed, which revealed nonspecific findings, with no evidence of viral cytopathic changes or malignancy. Because the first biopsy was non‐representative and given the patient's neurological history, a second biopsy was performed under sedation.

The histopathological analysis again showed nonspecific features and did not correspond to any inflammatory or neoplastic dermatosis. The tissue revealed a dense perivascular inflammatory infiltrate composed of lymphocytes and histiocytes, with prominent neutrophils forming microabscesses in the superficial dermis.

Given the two inconclusive histological results, the absence of an identifiable organic cause, and the clinical recurrence in a patient with neurological impairment who admitted to self‐inflicted lip biting, the findings were consistent with a diagnosis of factitious dermatosis (FC).

Once the diagnosis was confirmed, psychotherapy and psychopharmacological management were initiated to control anxious behaviors. Sertraline was started at 25 mg/day and progressively titrated to 200 mg/day, which was maintained under psychiatric supervision.

Topical treatment consisted of warm gauze soaked in saline solution applied to the affected areas to allow gentle decrusting. Mupirocin 2% ointment was then applied three times daily for 10 days. Following resolution of superficial cracking, tacrolimus 0.1% ointment was applied nightly for 2 months.

**FIGURE 2:**
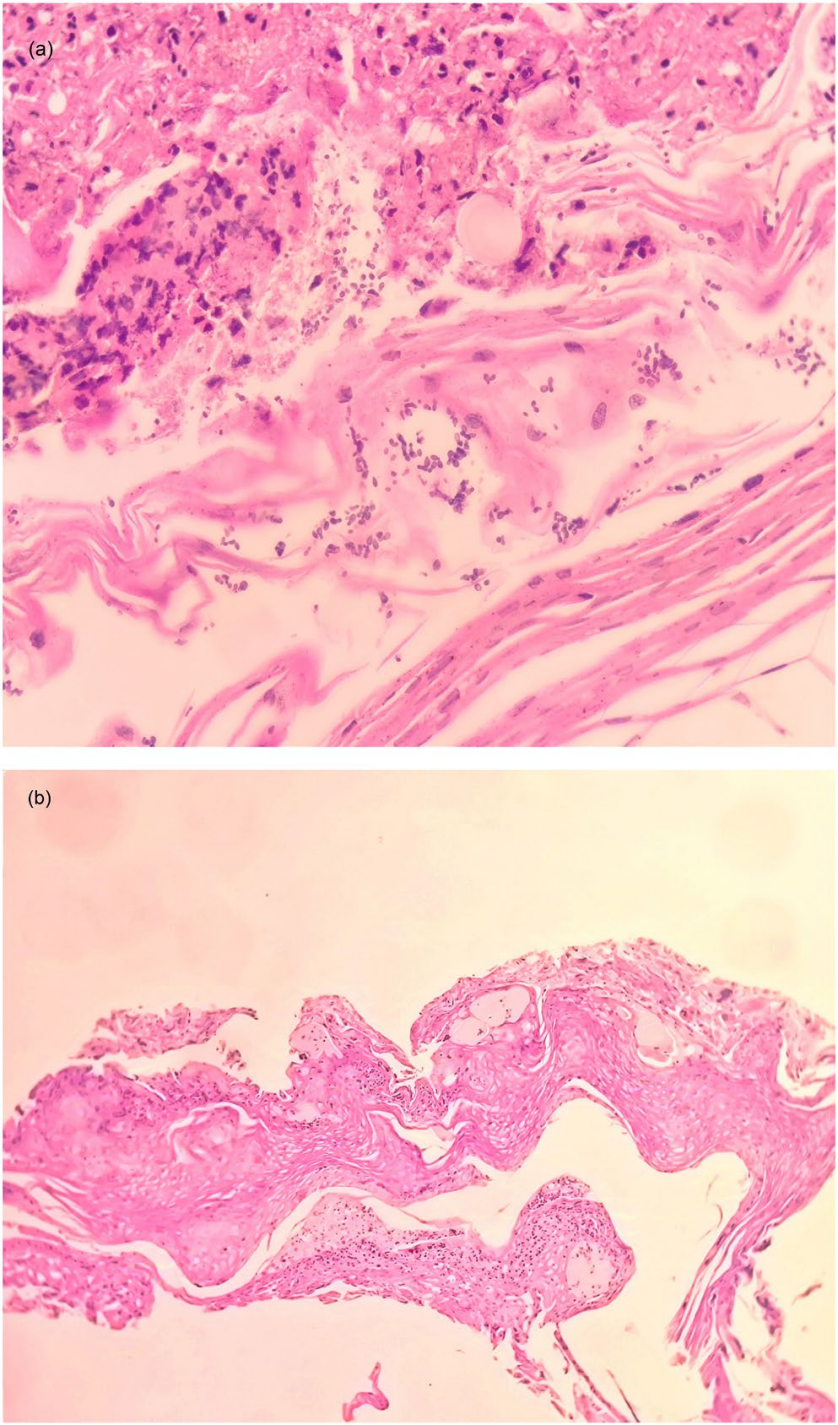
Case 1: (A, B) Hematoxylin and eosin staining. (A) This high‐magnification histological image (40×) reveals a dense perivascular inflammatory infiltrate composed of lymphocytes and histiocytes, with prominent neutrophils forming microabscesses in the superficial dermis. (B) At low magnification (10×), the image shows spongiosis, parakeratosis, and acanthosis in the squamous epithelium, along with focal ulceration. These features suggest chronic irritation and mechanical damage.

N‐acetylcysteine 600 mg was administered twice daily during the initial treatment phase. At the beginning of medical management, antibiotic coverage with amoxicillin/clavulanate 875/125 mg twice daily for 7 days was prescribed (Figure 2A,B).

### 2.3 Conclusion and Outcome

Based on the clinical history, the exclusion of neoplastic and infectious causes, and the patient's acknowledgment of self‐inflicted trauma, a final diagnosis of factitious dermatosis was established. The patient was managed with a multidisciplinary approach, involving dermatology, psychiatry, and neurology for behavioral and symptomatic control.

At follow‐up, no new lesions were observed, and the patient reported improvement in self‐injurious behavior after initiating psychological intervention. The total follow‐up period by dermatology was 6 months.

## 3 Case 2

### 3.1 Clinical History/Examination

**FIGURE 3:**
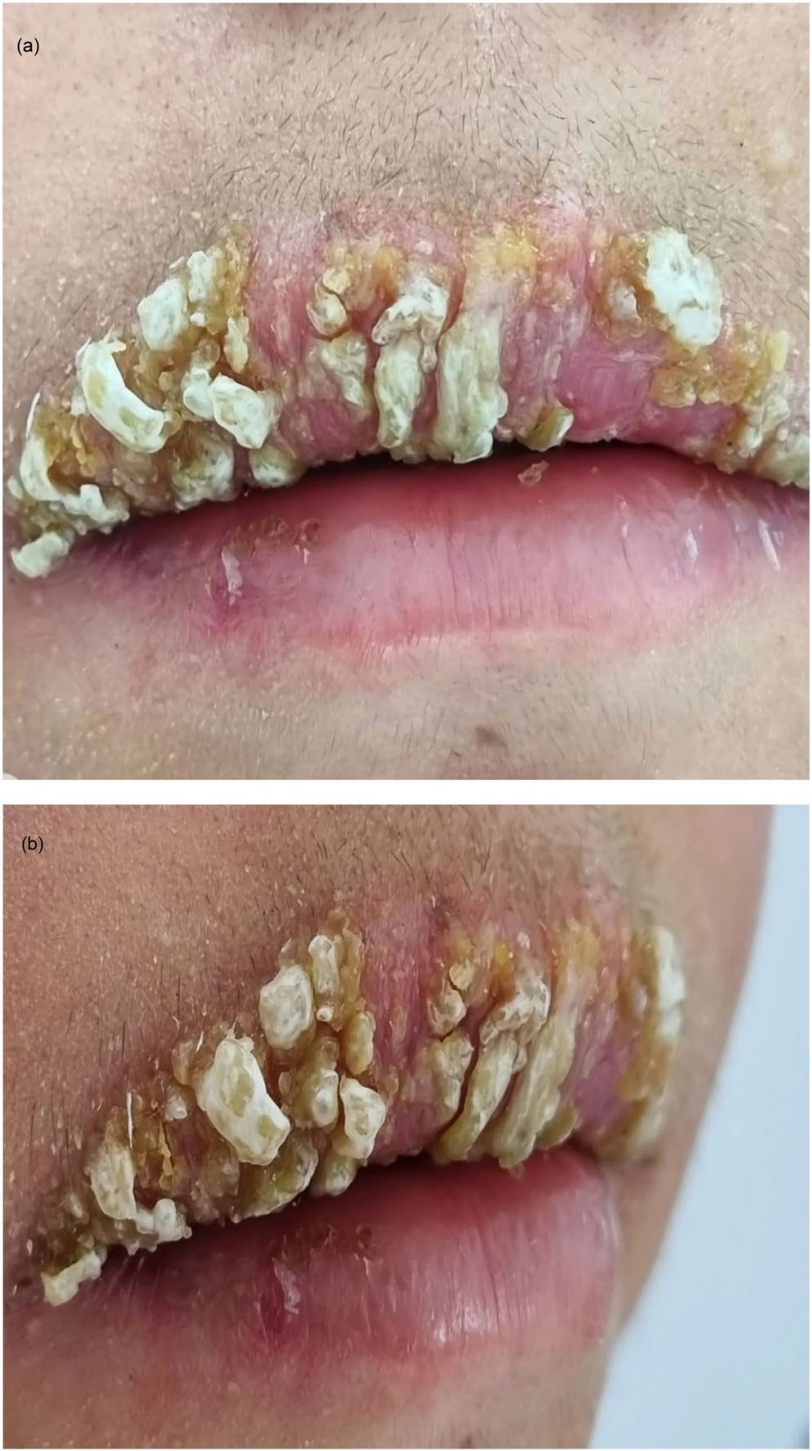
Case 2: (A, B) Multiple verrucous, erythematous‐appearing plaques on the upper lip.

A 24‐year‐old female patient with a history of anxiety disorder presented with a 1‐month history of a painful verrucous plaque on the upper lip, accompanied by erythema and scaling that interfered with eating and speaking. During the interview, the patient admitted to chronically traumatizing the epidermal layers of her lips using her teeth and fingernails, leading to constant microtrauma. She denied any prior medication use (Figure 3A,B).

### 3.2 Differential Diagnosis, Investigations, and Treatment

A skin biopsy revealed dense perivascular lymphohistiocytic dermatitis with neutrophilic infiltration forming subcorneal pustules in an epidermis showing spongiosis, acanthosis, and parakeratosis. Periodic acid–Schiff (PAS) staining was negative for fungal elements. While the histopathological pattern could suggest pustular cheilitis, the clinical findings—particularly the lesion distribution and the identification of repetitive self‐inflicted behavior—supported the diagnosis of FC.

**FIGURE 4:**
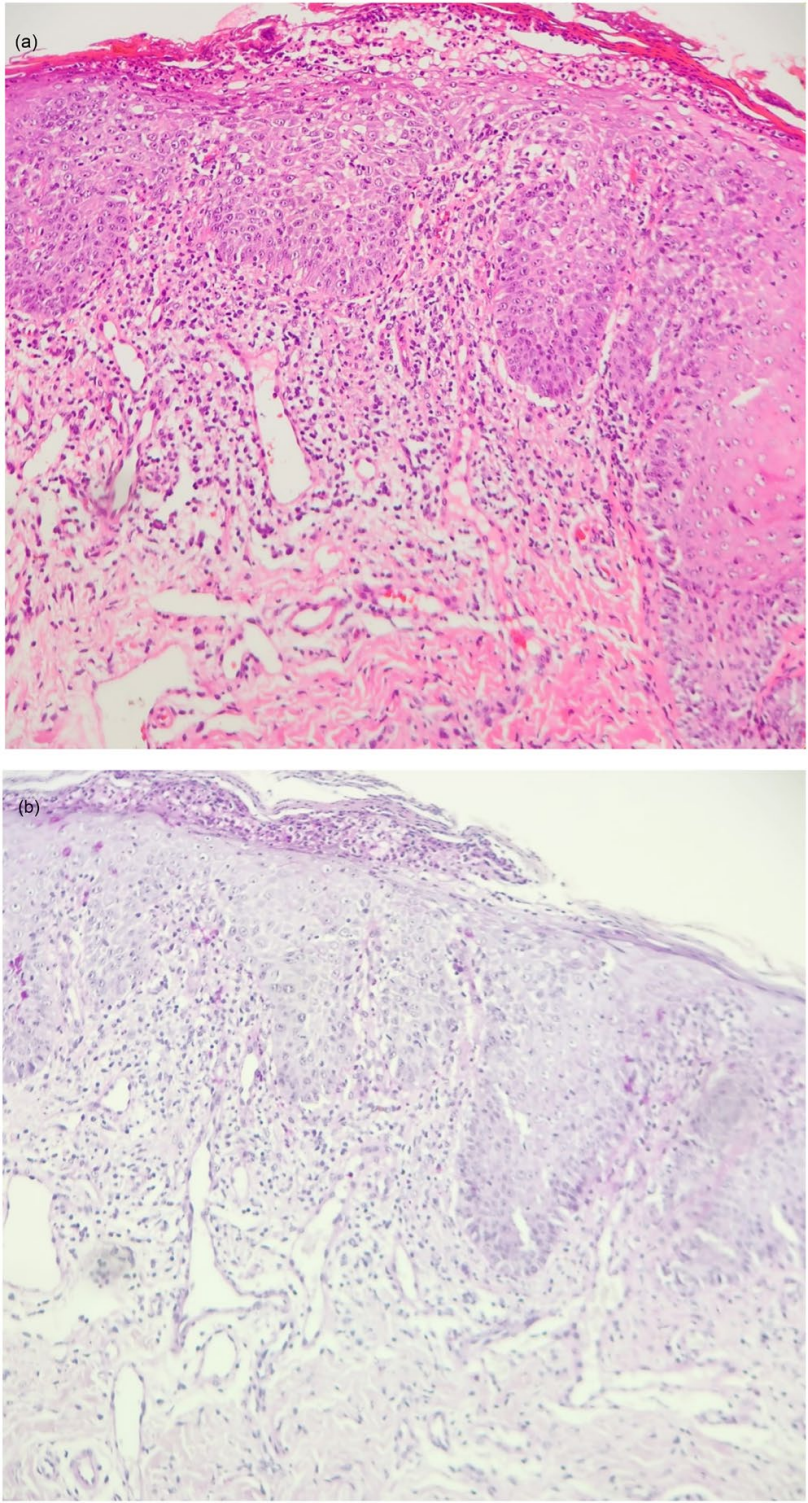
Case 2: (A, B) Hematoxylin and eosin stain. Dense perivascular lymphohistiocytic dermatitis with neutrophils permeating and forming subcorneal pustules in an epidermis showing spongiosis, acanthosis, and parakeratosis.

The same medical and topical therapy described in Case 1 was implemented. However, psychiatric management differed, as fluoxetine was initiated at 10 mg/day and progressively titrated to 60 mg/day, according to psychiatric indication and under follow‐up by the same specialty (Figure 4A,B).

### 3.3 Conclusion and Outcome

Based on the combination of clinical and histopathological findings, a diagnosis of FC was confirmed. The patient was referred for psychiatric evaluation and behavioral therapy, with subsequent improvement after addressing the underlying anxiety‐related behaviors. The total follow‐up period by dermatology was 6 months.

## 4 Discussion

Self‐induced dermatoses are divided into two main categories. The first includes impulsive and/or compulsive disorders that drive the behavior, such as trichotillomania, in which the patient is aware of the self‐inflicted nature and expresses a desire to stop. The second category encompasses factitious dermatoses, where the behavior is driven by unconscious psychological conflicts. A key distinguishing feature of this group is the patient's lack of recognition and unwillingness to cease the behavior. Factitious cheilitis, also known as morsicatio labiorum, belongs to this second group and clinically manifests as grayish‐white crusted plaques caused by repetitive licking, sucking, biting, or trauma [1].

The underlying behavior is often triggered by psychological, social, or domestic stressors and may serve as a coping mechanism or a way of seeking attention. Patients with FC frequently present with significant medical comorbidities that impact both quality of life and mental health. Moreover, a history of psychiatric disorders is common, including low self‐esteem, anxiety disorders, depression, obsessive–compulsive personality traits, other personality disorders, and, in some cases, delusional conditions involving the skin [2, 3].

Clinically, repetitive trauma presents as yellowish‐white crusted plaques on the vermilion of the lips—more commonly the lower lip—classically sparing the oral mucosa [3, 4]. These verrucous lesions are composed of dried saliva, cellular debris, keratin, and remnants of topical agents. Crust accumulation is common, particularly when patients avoid manipulation or cleaning of the affected area [5]. In severe cases, fissures and ulcerations may develop. Pain is often the predominant complaint and can be accompanied by pruritus or burning, significantly impairing eating, speaking, and mouth opening [6]. Secondary infections with *Staphylococcus aureus* or *Candida albicans* are common and may require topical or systemic antimicrobial therapy [3, 7].

Diagnosis is often delayed because lesions may resemble inflammatory or neoplastic disorders, leading to ineffective empirical treatments. A thorough medical history is essential, requiring a trusting physician–patient relationship to identify the underlying psychological or psychiatric disorder. A useful clinical clue for diagnosing FC is the removal of the compact crust with saline solution, revealing a normal‐appearing lip beneath [7].

Although histopathological examination is not mandatory, it is frequently performed due to diagnostic uncertainty. Typical findings include acanthosis of the mucosal epithelium with an irregular surface, associated with habitual lip biting. Basophilic debris, polymicrobial colonization, and reactive atypia in ulcerated lesions may appear, sometimes mimicking dysplasia [1, 3, 5]. Differential diagnoses include actinic cheilitis, glandular cheilitis, contact dermatitis, photosensitivity reactions, and neoplasms, with histopathology serving as a useful tool to distinguish among these entities [1].

To facilitate clinical assessment and guide the differential diagnosis—particularly in atypical cases or those overlapping with other forms of cheilitis—we present a schematic summary of the main differential diagnoses according to lesion site and morphology. This table aims to support systematic evaluation and recognition of self‐inflicted cases (Table 1).

**TABLE 1:** Differential diagnosis of factitious cheilitis.

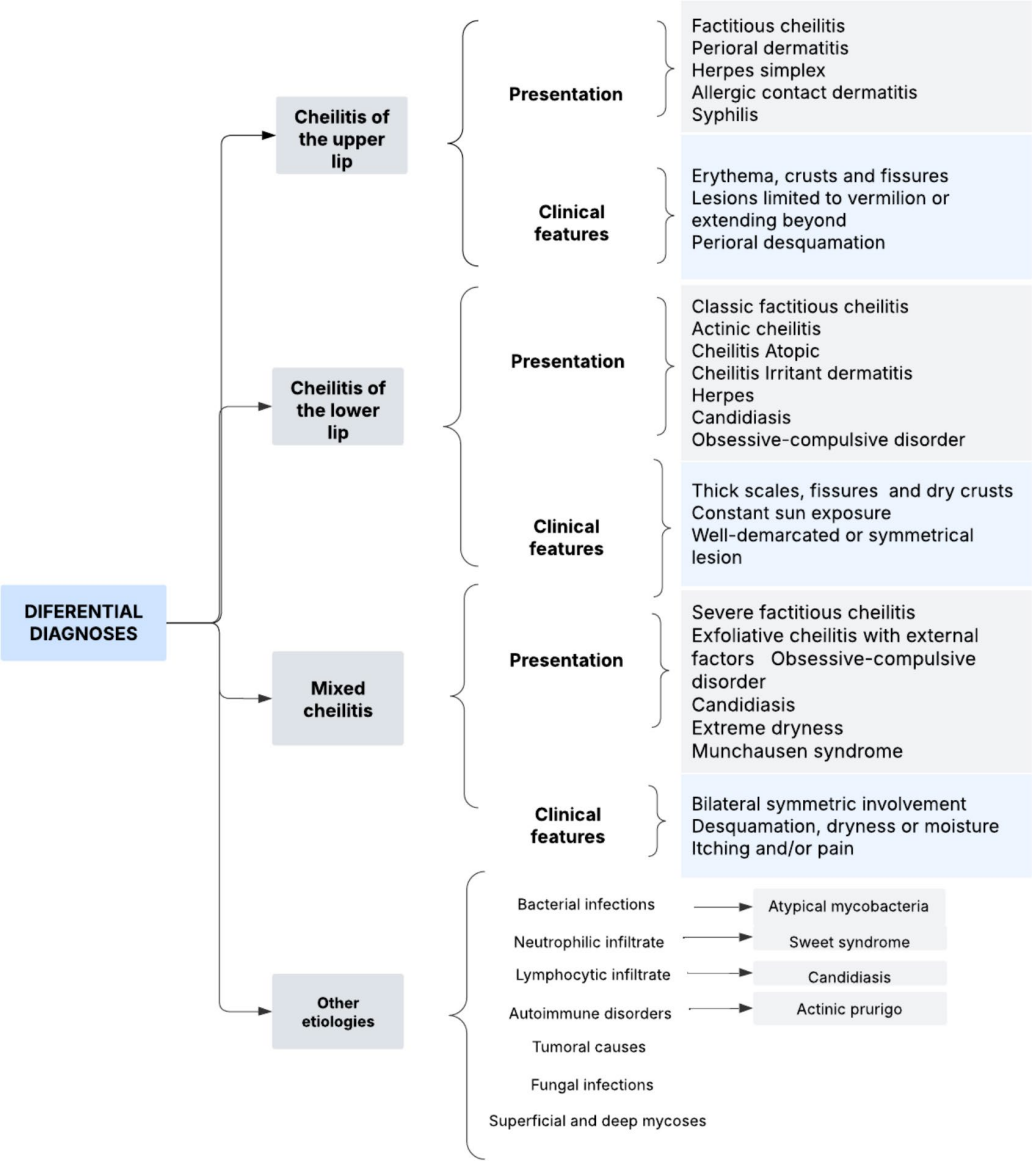

*Note:* Conditions affecting the upper lip include perioral dermatitis, herpes simplex, allergic contact dermatitis, and syphilis. Disorders involving the lower lip include actinic cheilitis, atopic cheilitis, irritant contact cheilitis, herpes infection, and candidiasis. Conditions affecting both lips include exfoliative cheilitis, candidiasis, severe xerosis, and Munchausen syndrome.

Management should be multidisciplinary, involving dermatology, psychiatry, psychology, oral pathology, and primary care [3]. Addressing the underlying psychological or psychiatric disorder is crucial. Direct confrontation regarding self‐inflicted behavior is discouraged; instead, the clinician should focus on identifying and managing the underlying stressors. Selective serotonin reuptake inhibitors (SSRIs), such as fluoxetine (starting at 10 mg, titrated up to 20–80 mg/day) and sertraline (starting at 25 mg, titrated up to 50–200 mg/day), have demonstrated efficacy in reducing compulsive behaviors. Atypical antipsychotics, including risperidone, olanzapine, and aripiprazole, have also shown utility, likely due to their modulatory effects on dopaminergic dysfunction [2].

Symptomatic topical therapies include 20% urea, topical corticosteroids, and—when secondary infection is present—antibiotics and antifungals. Treatment response is often limited, as many patients resist psychiatric or pharmacological interventions, and relapses due to psychological stress are frequent [1].

## 5 Conclusion

Factitious cheilitis is a rare and often underdiagnosed dermatosis with striking clinical features that may raise significant concern and diagnostic uncertainty for dermatologists. Empirical treatments and unnecessary diagnostic procedures contribute to substantial healthcare costs. Accurate diagnosis requires a detailed patient history, including psychiatric background. Effective management depends on a multidisciplinary approach that combines symptomatic dermatologic therapy with psychological interventions and, when appropriate, psychiatric medication.

## Author Contributions

**Daniel Mauricio Cuestas Rodriguez:** conceptualization, data curation, formal analysis, funding acquisition, investigation, resources, supervision, validation, visualization, writing – original draft. **Tatiana Carolina Reyes Vivas:** investigation, methodology, resources, supervision, validation. **Luis Daniel Pérez Cáceres:** data curation, formal analysis, investigation, resources, visualization, writing – original draft, writing – review and editing. **Laura Sofía Martínez Martínez:** investigation, methodology, project administration, supervision, writing – original draft, writing – review and editing. **Nahomy Giraldo Mejía:** conceptualization, investigation, methodology, writing – original draft, writing – review and editing. **Valentina Alvarez Mengual:** investigation, methodology, project administration, visualization, writing – original draft, writing – review and editing.

## Funding

The authors have nothing to report.

## Consent

Written informed consent was obtained from both patients for the publication of this case report and accompanying clinical images.

## Conflicts of Interest

The authors declare no conflicts of interest.

## Data Availability

The data supporting this case report are not publicly available due to patient privacy. De‐identified information can be provided by the corresponding author upon reasonable request.
